# Initial needle tracking with the first standalone combined infrared camera – CT system for brachytherapy—analysis of tracking accuracy and uncertainties

**DOI:** 10.1007/s00066-024-02253-3

**Published:** 2024-07-05

**Authors:** Andre Karius, Lisa Marie Leifeld, Vratislav Strnad, Claudia Schweizer, Rainer Fietkau, Christoph Bert

**Affiliations:** 1 Department of Radiation Oncology, Universitätsklinikum Erlangen, Friedrich-Alexander-Universität Erlangen-Nürnberg (FAU), Universitätsstraße 27, 91054 Erlangen, Germany; 2 Comprehensive Cancer Center Erlangen-EMN (CCC ER-EMN), Erlangen, Germany

**Keywords:** Needle navigation, Adaptive treatments, Optical tracking, Gynecologic brachytherapy, Patient-specific implantations

## Abstract

**Purpose:**

A prototype infrared camera – cone-beam computed tomography (CBCT) system for tracking in brachytherapy has recently been developed. We evaluated for the first time the corresponding tracking accuracy and uncertainties, and implemented a tracking-based prediction of needles on CBCT scans.

**Methods:**

A marker tool rigidly attached to needles was 3D printed. The precision and accuracy of tool tracking was then evaluated for both static and dynamic scenarios. Euclidean distances between the tracked and CBCT-derived markers were assessed as well. To implement needle tracking, ground truth models of the tool attached to 200 mm and 160 mm needles were matched to the tracked positions in order to project the needles into CBCT scans. Deviations between projected and actual needle tips were measured. Finally, we put our results into perspective with simulations of the system’s tracking uncertainties.

**Results:**

For the stationary scenario and dynamic movements, we achieved tool-tracking precision and accuracy of 0.04 ± 0.06 mm and 0.16 ± 0.18 mm, respectively. The tracked marker positions differed by 0.52 ± 0.18 mm from the positions determined via CBCT. In addition, the predicted needle tips in air deviated from the actual tip positions by only 1.62 ± 0.68 mm (200 mm needle) and 1.49 ± 0.62 mm (160 mm needle). The simulated tracking uncertainties resulted in tip variations of 1.58 ± 0.91 mm and 1.31 ± 0.69 mm for the 200 mm and 160 mm needles, respectively.

**Conclusion:**

With the innovative system it was possible to achieve a high tracking and prediction accuracy of marker tool and needles. The system shows high potential for applicator tracking in brachytherapy.

## Introduction

Image-based adaptive brachytherapy has become a clinical standard for many brachytherapy workflows [1–5]. The terms “image-based” and “adaptive” refer to the use of particularly three-dimensional (3D) imaging such as computed tomography (CT) to visualize the patient’s anatomy with or without applicators in situ, and the desire and possibility to adapt treatments to the individual case based on these images. This includes the actual implantation [5, 6] as well as quality assurance during the treatment course [4, 7–9].

For implantations, the goal is to create an optimal applicator arrangement for treating the specific target volume. Respective approaches are currently a hot research topic, especially in gynecologic brachytherapy [2, 7, 10–12] to treat diseases extending into the parametrium or deep in the pelvis with combined intracavitary and interstitial techniques [13–17]. In these cases, ultrasound alone may not be suitable for adequate visualization of the anatomy of interest. The use of CT or magnetic resonance imaging (MRI) for this purpose allows implant adjustments based on acquired images, but not real-time guidance with immediate feedback on the exact applicator path in situ at any time during the interventional procedure. However, such guidance seems beneficial, particularly for implantations close to organs at risk to avoid injuries [7, 18]. Furthermore, it is desirable to reduce the need for repetitive image acquisitions to save time and effort and reduce patient exposure.

For this reason, we aim to track applicator needles fixed to a tracking tool, which consists of multiple infrared (IR) markers, with IR cameras. Given a rigid relation between the markers and the needle tip, tracking the tool (which is located at the distal parts of the applicator outside the patient) can be used to predict the course of the needle in situ in real time. This course can then be displayed, e.g., on one CT scan acquired at the beginning of the implantation procedure. For this purpose, we implemented a standalone combined IR camera – cone-beam CT (CBCT) system for the first time, as described previously [18]. However, a detailed analysis of the needle-tracking accuracy and uncertainties achievable with this system has not yet been published.

Therefore, the aim of this work was to establish a complete tracking workflow, including definition of a marker tool rigidly attachable to needles, to assess the corresponding tracking accuracy and to predict the needle tips on CBCT scans. As prediction reliability is of utmost importance to ensure accurate implantations, an estimation of the corresponding tracking uncertainties was also performed. Our analysis thus provided a basis for the clinical implementation of such tracking, in order to enable real-time needle guidance even in cases where ultrasound imaging is not appropriate. Since the assumption of a rigid tool–applicator relation is a prerequisite for the workflow, the investigations were limited to metal needles.

## Materials and methods

### Combined system and tracking tool

**Fig. 1:**
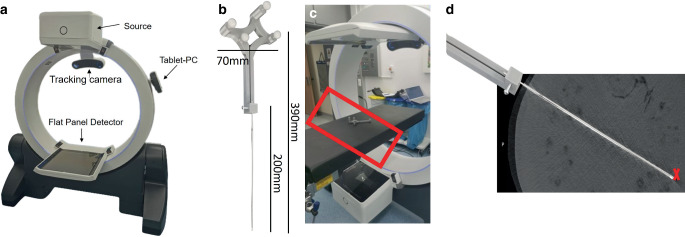
Shown is the standalone combined system integrating a tracking camera directly into the mobile ImagingRing m (**a**). For implementing needle tracking, we created a corresponding tool of the indicated dimensions (**b**), which was placed in the main operation area (*marked red*, **c**) for future clinical settings. For evaluating tracking accuracy, we inserted needles attached to the tool into a melon and compared the actual needle tip to the predicted (*marked red*) needle tip (**d**)

In this work, we used a prototype of a standalone combined camera–CBCT system (Fig. 1a), which integrates two IR cameras into a mobile CBCT scanner. A first technical characterization of the system has been reported previously [18]. In summary, two Prime^x^ 13W IR cameras (OptiTrack, Corvallis, OR, USA) were integrated into the mobile CBCT scanner ImagingRing m (medPhoton, Salzburg, Austria), which is well suited for imaging in the lithotomy position [7, 19, 20]. The cameras emit IR light and receive corresponding reflections, e.g. from passive IR markers, within a field of view (FOV) of 82° in the horizontal and 70° in the vertical direction.

High-quality calibration and camera–CBCT registration are prerequisites for robust tracking. The optimal calibration and fusion procedures for achieving the system’s maximum performance of 0.46 ± 0.28 mm accuracy (for transferring marker positions from the camera into the CBCT coordinate system) have been reported previously [18]. These optimal procedures were also applied to the device prior to the present work. The purpose of this study was to evaluate for the first time the needle tracking and prediction accuracy achievable with the new system.

To predict needle courses on CBCT scans, a rigidly attachable tracking tool was created. The intention was that by tracking this tool fixed to the distal part of a needle and transferring the tool position into the CBCT coordinate system, the corresponding needle tip could be projected into a previously acquired CBCT scan due to the rigid relationships. Our tool consisted of four IR markers (12.7 mm diameter) attached at different heights and distances to ensure asymmetry of the configuration (Fig. 1b). In addition, a clamp allowed a needle to be fixed in position by tightening it with a screw, which eliminated any uncertainties regarding the exact needle–tool connection. Because of the resulting constant offset between the clamp and the markers, the setup appeared suitable for determining needle courses via tracking. In general, the tool was designed to have two finger recesses on the bottom to hold it during implantation. It could either be attached to an already inserted needle or attached to the needle before or during insertion to allow real-time tracking of the dynamic process. The tool was designed using Tinkercad (Autodesk, San Rafael, CA, USA) with the dimensions shown in Fig. 1b and printed on a 3D printer using polyactide (PLA) filament. In the present work, the tool was attached to 18G Trocar point titanium needles (Elekta, Netherlands) of lengths 200 and 160 mm, which represent the longest and median-length needles used in our standard gynecologic brachytherapy workflows.

### Implementation of needle tracking

To implement needle tracking, ground truth models of the tool attached to the studied needles of 200 and 160 mm length were created. Each needle was clamped into the tool and a corresponding CT scan with 0.3 × 0.3 × 1 mm^3^ voxel size was acquired using a SOMATOM go.Open Pro scanner (Siemens Healthineers, Forchheim, Germany). This conventional CT scanner was chosen for establishing the ground truth due to an improved handling of scatter and beam hardening potentially caused by the IR markers compared to the ImagingRing CBCT. Needle course and marker positions were then determined by thresholding and applying the MATLAB (MathWorks, Natick, MA, USA) “regionprops” function [21], which resulted in the desired ground truth model.

In the context of IR tracking, the position of the needle tip can only be deduced via its rigid relationship to the attached tool, but cannot be directly detected by the cameras due to a missing line-of-sight during/after needle insertion. Therefore, our approach was to create a match of the ground truth tool to the marker positions determined by corresponding IR tracking (note that the tracked marker positions were already available in the CBCT coordinate system due to the previously described [18] camera–CBCT registration). Matching was performed according to the rigid transformation principle [22] implemented in MATLAB (MathWorks, USA). In this principle, the four tracked and ground truth 3D marker positions were each considered as 4 × 3 matrices *A* and *B*, respectively, where *a* and *b* represent the corresponding centroids of both point clouds. The optimal transformation between the two clouds was determined by computing the covariance *cov* and its singular value decomposition *SVD*:

$$\begin{array}{c} cov={\sum }_{i=1}^{4}\left(B_{i}-b\right)\left(A_{i}-a\right)^{T},\\ \left[U,S,V\right]=SVD\left(cov\right). \end{array}$$

This yielded the best-fit rotation *r* and translation *t* for matching the ground truth *B* onto the tracked markers by $B'=r\cdot B+t$, where

$$r=VU^{T},\quad t=a-r\cdot b.$$

By applying this transformation to the ground truth model, including the rigidly attached needle, it became possible to project the needle course and especially the needle tip location into the CBCT coordinate system.

### Analysis of tool tracking

To analyze tool tracking for a stationary scenario, the tool was placed in five different positions within the area where its main operation will be performed in future clinical settings (indicated by the red rectangle in Fig. 1c). At each position, the markers were tracked every 2 s for 5 min. For each snapshot and marker, the positional differences from the individual mean marker positions were calculated. In a second step, to evaluate the effect of dynamic movements, the tool was moved randomly by hand within the camera FOV twice for 8 min. Tracking was performed every 2 s. For each snapshot, the distances between the individual markers were measured and compared with the corresponding distances obtained for the stationary scenario mentioned above. In this way, the inaccuracies introduced by tool movements were determined.

In addition, the agreement of the tracked marker positions (which were already available in the CBCT coordinate system due to the previously described [18] camera–CBCT registration) with the positions determined directly on CBCT scans was investigated. For this purpose, the tool was placed at 10 different locations within the overlapping camera and CBCT FOV. Note that in clinical scenarios, the patient has to be positioned within the CBCT FOV and, therefore, there would be no space to place the tool here as well. However, our procedure was the only way to truly compare tracked markers and markers identified directly via CBCT. At each location, tracking was performed every 2 s for 10 s, and the average marker positions were determined within the CBCT coordinate system. Additionally, CBCT scans with a voxel size of 0.3 × 0.3 × 1 mm^3^ were acquired and the marker centroids were calculated by thresholding and applying the MATLAB (MathWorks, USA) “regionprops” function [21]. The positions obtained in both ways were compared by calculating the corresponding Euclidean distances as a measure of tracking accuracy.

### Analysis of needle tip predictions

To evaluate the accuracy of needle tip prediction, the tool with the examined needle clamped (we investigated titanium needles of 200 mm and 160 mm length, as mentioned above) was placed at 10 different positions within the area where its main operation will be performed in future clinical settings (Fig. 1c). At each position, it was tracked every 2 s for 10 s. The respective ground truth was the matched according to the section “Implementation of needle tracking” to the averaged tracked marker positions in order to project the needle course into the CBCT coordinate system. In this way, the corresponding needle tip location was predicted. The Euclidean distance between the matched and averaged positions was calculated as a measure of the match quality.

In addition, a CBCT scan (voxel size of 0.3 × 0.3 × 1 mm^3^) of the region around the needle tip was acquired for each examined position. On this scan, the needle was manually reconstructed using the treatment planning system Oncentra Brachy (Nucletron, Veenendaal, Netherlands). The Euclidean distance between the predicted and reconstructed needle tip was calculated, both in total and separately for the direction transverse and longitudinal to the needle course.

Two sets of measurements were performed. First, the clamped needles were suspended freely in air. Second, the needles were inserted into a watermelon (Fig. 1d) to simulate implantations potentially associated with needle bending.

### Assessment of propagated tracking uncertainties

As a last step, it was investigated whether the uncertainties in needle tip prediction determined in this work were caused by our experimental setup or were mainly due to the tracking system itself. In addition, our aim was to theoretically determine the uncertainties to be expected in the prediction of the needle tips in clinical practice (without taking needle bending into account). It should be noted that the uncertainty analysis did not address potential effects of needle bending during implantation, as these are considered to be highly operator dependent, and our goal was to assess the maximum prediction accuracy achievable with the new system.

To accomplish this, we performed a simulation of the propagation of individual tracking uncertainties to obtain an estimate of the needle tip prediction uncertainty. First, note that the distance between the camera and the main clinical operation area is about 1.5-times larger than the distance set for the performed camera–CBCT marker comparison (Sect. Analysis of tool tracking), where the tool was placed within the CBCT FOV. This could, in the worst case (i.e., in the case of rotational camera–CBCT registration errors), lead to up to 1.5-times larger positional shifts (compared to the results of Sect. Analysis of tool tracking) between tracked and actual marker locations. This increased tracking uncertainty will be referred to as *µ*_*a*_ *±* *σ*_*a*_ in the following.

Second, we previously investigated the spatial fidelity of the camera coordinate system that can be achieved by its corresponding calibration [18]. The optimal mean calibration accuracy was determined to *µ*_*b*_ *±* *σ*_*b*_ = 0.18 ± 0.03 mm, which will also be propagated as respective uncertainty in the tracking workflow. Third, the tracking itself features a statistical position uncertainty *µ*_*c*_ *±* *σ*_*c*_, which was experimentally determined in Sect. Analysis of tool tracking as well.

To provide a conservative estimate of the total uncertainties to be expected in needle tip predictions, we considered a propagation of all three individual uncertainties. For this purpose, the ground truth model of the examined needle clamped in the tool was shifted by a random distance (taken from a Gaussian distribution with mean µ_a_ and standard deviation σ_a_) in an arbitrary spatial direction. This was done because a camera–CBCT registration error affects all markers approximately the same due to the small tool extent compared to the tool–camera distance. Each marker was then individually shifted in random directions considering another Gaussian distribution (mean µ_b_, standard deviation σ_b_) to account for the calibration inaccuracies and a Gaussian distribution (µ_c_, σ_c_) to account for the tracking uncertainty. This sequence of three shifts was simulated a total of 100,000 times. The ground truth tool model (including the rigidly attached needle) was then matched to each simulated tool according to Sect. Implementation of needle tracking. The Euclidean distances between the resulting and original needle tips were computed in total and separately for the direction transverse and longitudinal to the original needle course. This served as a measure of the needle tip prediction uncertainties inherent to the combined camera–CBCT system.

## Results

### Tool tracking

**Fig. 2:**
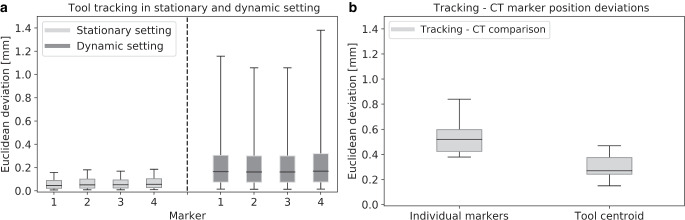
Shown are the Euclidean deviations between tracked marker positions and mean marker positions calculated for each tool marker to evaluate tracking precision in the stationary setting. The Euclidean distance deviation between the individual markers obtained when comparing tracking and the ground truth in the dynamic measurements is also provided (**a**). Moreover, the Euclidean distances of tracked marker positions and positions determined directly via CT as well as the distances of the corresponding tool centroid are illustrated (**b**). In the *boxplots*, the *horizontal lines* indicate the median, the *boxes* the interquartile range, and the *whiskers* the 95th percentile. Outliers are not shown for clarity of illustration

The tracking precision achieved in the stationary scenario is shown in Fig. 2a. Considering all markers, the median deviation between measured and averaged positions was 0.04 ± 0.06 mm (median ± standard deviation). Maximum outliers were up to 0.48 mm, i.e. all uncertainties were in the submillimeter range. Regarding the dynamic setting, we measured (averaged over all snapshots) median marker distance deviations of only 0.16 ± 0.18 mm (Fig. 2a). However, a maximum deviation of 16.3 mm was observed as well, which highlighted the need to specifically account for dynamic movements during tracking.

The comparison of the tracked and CBCT-imaged marker positions revealed median Euclidean deviations of 0.52 ± 0.18 mm (maximum: 0.95 mm) for all individual markers and 0.27 ± 0.19 mm (maximum: 0.82 mm) for the tool centroid within the CBCT coordinate system (Fig. 2b). Hence, the obtained inaccuracies were larger than the tracking precision reported above and thus related to the camera–CBCT fusion process.

### Needle tip predictions

For needle tip predictions, the ground truth of the tracking tool was matched to the tracked marker positions according to Sect. Implementation of needle tracking, and the corresponding Euclidean distances were computed as a measure of the match quality. Considering all air and melon measurements performed, we obtained median Euclidean distances between tracked markers and matched ground truth of 0.18 ± 0.06 mm. Maximum deviations of 0.26 mm were found.

**Fig. 3:**
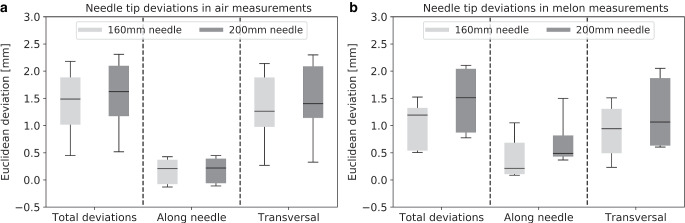
Shown are the Euclidean deviations between predicted needle tips and actually reconstructed needle tips, both totally as well as separated by direction along the needle and transverse to it. The results are provided for the air (**a**) and melon (**b**) measurements. In the *boxplots*, the *horizontal lines *indicate the median, the *boxes* the interquartile range, and the *whiskers* the 95th percentile. Outliers are not shown for clarity of illustration

In the air measurements, the predicted needle tips showed median deviations of 1.62 ± 0.68 mm (200 mm needle) and 1.49 ± 0.62 mm (160 mm needle) from the actual reconstructions (Fig. 3a). For the 200 mm needle, deviations along the needle direction had a median of 0.22 ± 0.30 mm (maximum: 1.13 mm), while transverse variations amounted to 1.40 ± 0.74 mm (maximum: 2.41 mm). For the 160 mm needle, similar results of 0.21 ± 0.30 mm and 1.27 ± 0.67 mm were obtained for the longitudinal and transverse directions, respectively.

Regarding the melon study, the predicted tips showed median discrepancies from the reconstruction of 1.51 ± 0.76 mm and 1.19 ± 0.55 mm for the 200 mm and 160 mm needle, respectively (Fig. 3b). Maximum variations were up to 3.0 mm (200 mm needle). Separated by direction, the deviations along the needle course were smaller, with medians of 0.49 ± 0.48 mm and 0.22 ± 0.48 mm for the 200 mm and 160 mm needle, respectively, than the variations transverse to the needle course of 1.06 ± 0.75 mm and 0.94 ± 0.60 mm.

### Assessment of tracking uncertainties

According to the results described above, the parameters of the Gaussian distributions used to simulate the tracking uncertainties were determined to be *µ*_*a*_ *±* *σ*_*a*_ = 0.85 ± 0.27 mm and *µ*_*c*_ *±* *σ*_*c*_ = 0.16 ± 0.18 mm. The latter was chosen to account for the imprecisions found in the dynamic tool measurements, since these exceeded those of the stationary scenario (Sect. Tool tracking). Based on this and averaged over all simulations, we obtained median Euclidean position deviations of 0.19 ± 0.10 mm (90% confidence interval [0.06 mm; 0.36 mm]) in matching the ground truth tool to the simulated marker positions.

**Fig. 4:**
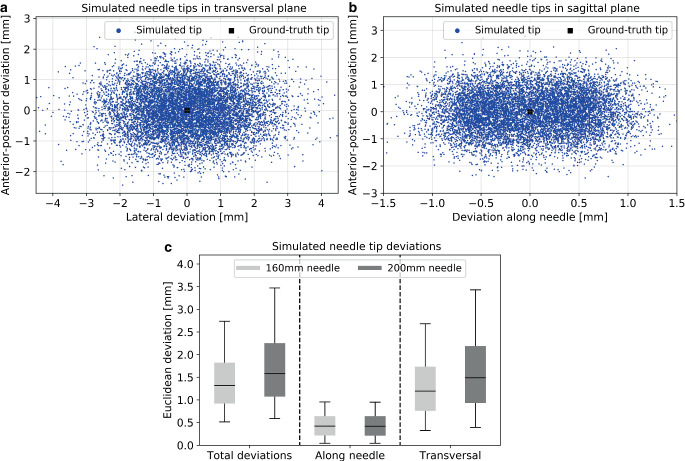
Shown are the Euclidean deviations between the ground truth needle tip (*black*) and the simulated needle tip (*blue*) considering the 160 mm needle for the plane transverse to the needle course (**a**) and the sagittal plane along the needle (**b**). The corresponding total Euclidean deviations between the tips as well as the deviations along the needle directions and transverse to it are provided for the 200 mm and 160 mm needle as well (**c**). In the *boxplots*, the *horizontal lines *indicate the median, the *boxes* the interquartile range, and the *whiskers* the 95th percentile. Outliers are not shown for clarity of illustration

The observed deviations (separated by spatial directions) between simulated and actual needle tips are shown exemplarily for the 160 mm needle in Fig. 4a, b. The simulations yielded median deviations between actual and predicted needle tips of 1.58 ± 0.91 mm ([0.58 mm; 3.49 mm]) for the 200 mm needle and 1.31 ± 0.69 mm ([0.51 mm; 2.73 mm]) for the 160 mm needle (Fig. 4c). Separated by direction, we obtained deviations of 0.41 ± 0.29 mm ([0.04 mm; 0.95 mm]) in the longitudinal direction and 1.49 ± 0.95 mm ([0.39 mm; 3.45 mm]) in the transverse direction for the 200 mm needle. For the 160 mm needle, the longitudinal and transverse deviations were 0.42 ± 0.29 mm ([0.04 mm; 0.95 mm]) and 1.19 ± 0.73 mm ([0.32 mm; 2.68 mm]), respectively (Fig. 4c). The uncertainties in the prediction of the needle tips were therefore substantially higher than the individual uncertainties of the tracking system used as the starting point in the simulation, since the length of the needle represented a corresponding uncertainty multiplier.

## Discussion

In this work, we investigated for the first time the applicator tracking accuracy achievable with a novel standalone camera–CBCT system. We achieved a median accuracy of needle tip prediction and marker determination of up to 1.49 ± 0.62 mm and 0.52 ± 0.18 mm, respectively. This is considered high compared to optical tracking approaches using external cameras (about > 1.6 mm) [23–27]. For instance, Zhang et al. [25] achieved a median surgical navigation accuracy of 4.1 mm using iPlan CMF (Brainlab, Germany). Ji et al. [26] determined needle tips in prostate brachytherapy using the IGS-MO Optical Image Navigation system (Xinbo Medical Technology, China) with an accuracy of 3.1 ± 1.8 mm. A phantom study by Krempien et al. [27] using a marker tool attached to the tip end regions of needles and an external Polaris camera (Northern Digital, Waterloo, Ontario, Canada) yielded an average tip deviation from prediction of 1.6 mm. The main difference between these approaches and the tracking performed in the present work was our rigid connection of the IR camera to the CBCT scanner. Therefore, the need to track both the CBCT scanner and the tool with the camera to enable image-guided navigation was reduced to tracking the tool only. Furthermore, it should be noted that our long needles can easily be affected by even small tool-tracking errors, and thus the reported accuracies need to be put into perspective when comparing with other studies [23–27]. Our work thus showed the high potential of the camera–CBCT system for accurate tracking. Nevertheless, further improvements may be achieved through continued development in the near future.

Corresponding improvements can be achieved in particular with respect to camera calibration and the camera–CBCT fusion, as described previously [18] (the fusion error was the largest uncertainty considered in our simulations and represented the main issue to be addressed), as well as by developing the tracking procedure itself. For instance, we only implemented tracking based on the rigid transformation principle because it has been shown to be very robust for a small number of points at high computational speed [22]. When matching the ground truth tool to the tracked marker positions, we obtained low marker deviations of only 0.18 ± 0.06 mm. However, it has not yet been investigated whether other registration approaches might offer higher fusion and tracking accuracy. Further optimization potential also lies in the tool design. In this work, we created a 3D tool based on our experience with the combined system, but possible benefits of different designs with different numbers and/or arrangements of markers should be investigated. In particular, a larger number of markers may result in positional tracking uncertainties having less impact on the overall tracking quality and, thus, needle predictions. On the other hand, the tool must meet all hygienic requirements while remaining robust and ergonomic. For clinical operation, requirements regarding sterility of the tool will have to be defined and fulfilled.

The tracking precision obtained for both the static and dynamic scenarios was generally high and in the submillimeter range. However, strong outliers were observed as well, especially in the dynamic setting. Since the tool was moved randomly for these measurements, the exact causes of these outliers could not be identified. However, they could be attributed to partial shielding of the markers, incorrect marker assignments, or movements that might have been too fast, which may have caused marker blurring during the measurement. In order to achieve high accuracy, it was therefore recommended to ensure slow and smooth movements of the tool during needle insertion to enable adequate navigation. To account for tracking uncertainties due to single marker position deviations, a higher number of markers with software-sided automatic addressing of such outliers may be beneficial.

In addition to empirical tracking accuracy, we also determined tracking uncertainties. These were calculated to a median of 1.31 ± 0.69 mm for the 160 mm needle and provide accuracy thresholds with which tracking can be reasonably performed with the current system state. In the empirical measurements, we obtained needle tip deviations from the prediction that were consistent with our simulations, thus confirming their validity. However, in clinical scenarios, even more uncertainties may have to be considered. For example, needles and/or the tool may bend during implantations [28–30], resulting in varying deviations from the prediction, since the assumed rigid relation between the tracking tool and the needle course would no longer be valid. This is also the reason why our approach is limited to the use of rigid applicators such as metal needles and is not applicable to flexible plastic catheters. Needle bending was not observed in the melon study (although the hardness of its rind simulated tissue resistance) and was also not investigated in the present work, as it is considered to be highly operator dependent based on clinical experience. However, our goal was to assess the maximum needle prediction accuracy achievable with the new system, regardless of any limitations caused by the operator. To the best of our knowledge, investigations on the actual extent of needle bending during implantations for gynecologic brachytherapy have not been performed so far and remain the subject of future clinical studies. Potential effects could be corrected by using radiography (also provided by the ImagingRing) or by identifying the needle tip location on ultrasound after its insertion to a certain depth and using the proposed IR tracking only for the last few centimeters to be implanted (in areas where ultrasound imaging allows no adequate visualization of the anatomy). The latter procedure is our current proposal for handling the occurrence of needle bending, but its applicability and feasibility have to be investigated in further studies quantifying the extent and clinical impact of bending in situ and assessing the actual clinical necessity of bending corrections. As a further uncertainty, potential limitations of the threshold-based ground truth determination based on CT scans, e.g. due to partial volume effects [31, 32], need to be quantified and reduced. On the other hand, reconstruction uncertainties have to be considered when reporting needle deviations as well. For high-contrast applicators, a respective mean interobserver variability in the range of 0.6–0.9 mm has been reported [33–36], which may contribute to the found needle tip deviations. According to these descriptions, all uncertainties and risks associated with the tracking procedure will have to be weighed against its benefits prior to clinical use.

In addition, it should be noted that our needle-tracking investigations were still based on an offline ground truth matching in MATLAB. For real-time guidance, the matching would have to be an integral software component of the system, allowing appropriate needle predictions within milliseconds. This must be the focus of future work, as it is the only way to ensure real-time feedback on applicator positions at any point in the procedure. This is a major advantage of our tracking approach over image-based (but not image-guided) implantation using 3D imaging only and will allow implantations to be performed in the sense of true image-guided adaptive brachytherapy. This will improve the ability to optimize the dose distribution in the subsequent treatment planning process and adapt the treatment to the individual patient with a high degree of accuracy.

Our focus for future clinical applications of the system will be on its use for interstitial gynecologic brachytherapy to enable accurate implantations in anatomical regions where ultrasound guidance alone is not sufficient [13–17]. Promising applications regarding improved needle placement in prostate brachytherapy [26] may arise as well. For gynecologic brachytherapy, multiple intraoperative CBCT scans in a lithotomy patient position can be necessary (depending on the case) to verify and adapt applicator and needle positions for creating a sufficient implant arrangement. This is associated with effort for medical staff as well as additional dose exposure to the patient [18]. With the implementation of the new device into clinical routine, the aim is to acquire only one CBCT scan at a certain time during surgery, to project the needle course in situ during the subsequent insertion process in real time into this scan based on the optical tracking, and in this way to position the needle as intended.

However, it has to be mentioned that the aim of the present work was an initial preclinical analysis of the accuracy and uncertainties of the tracking procedure and system, whereas the clinical applicability and feasibility of the proposed workflow will have to be investigated in further examinations. For instance, we currently plan to work with one single marker tool and to attach it to one needle, to insert the latter guided by the tracking procedure, and to then detach the marker tool from this needle and start with the next one. This is because with the current tool design, multiple marker tools could only be attached to multiple needles (e.g., one tool to each needle of an implant) without colliding if the needles were spaced > 5 mm in both anteroposterior and lateral direction. In case of a smaller spacing being required, adaptions of the marker tool design would be required. However, from a solely technical perspective, it would be feasible to track multiple tools simultaneously. Therefore, the establishment of tracking-guided brachytherapy implantation workflows forms a promising topic of research, aiming at improvement of implantation accuracy.

## Data Availability

The data that support the findings of this work are available from the corresponding author upon reasonable request.
